# Severe Gingival Enlargement Associated With Methamphetamine Abuse: A Case Report

**DOI:** 10.7759/cureus.73757

**Published:** 2024-11-15

**Authors:** Eliane Porto Barboza, Steven J Caldroney, Beatriz Panariello, Diogo M Rodrigues, William Bell

**Affiliations:** 1 Dentistry, Lake Erie College of Osteopathic Medicine, Bradenton, USA; 2 Periodontics, National Institute of Dental Science (INCO25), Niteroi, BRA

**Keywords:** advanced periodontitis, drug and substance abuse, drug-influenced gingival enlargement, microbiology, oral microbiology, periodontitis

## Abstract

Gingival enlargement (GE) is characterized by the overgrowth of gingival tissues, often exacerbated by dental biofilm and certain medications or substances. This report presents a case of severe GE in a 61-year-old male with generalized stage 4, grade C periodontitis linked to methamphetamine use, which the patient disclosed occurred during a period of homelessness. The treatment involved antibiotic therapy, extractions, gingivectomy, gingivoplasty, and osteoplasty to facilitate future oral rehabilitation. After treatment, the patient reported no pain and improved eating habits, significantly enhancing his quality of life. This case highlights the critical relationship between GE, substance abuse, and advanced periodontal disease, underscoring the need for increased awareness and interdisciplinary approaches in managing such patients.

## Introduction

Gingival enlargement (GE) encompasses a range of conditions characterized by the overgrowth of gingival tissues, leading to an increase in the size of the keratinized gingiva [1]. One specific type of GE is drug-influenced, which is categorized under dental biofilm-induced gingivitis [2]. For drug-influenced gingival overgrowth to happen, dental biofilm must be present alongside the use of medications known to trigger this condition, such as phenytoin, nifedipine, cyclosporine [1], or the abuse in the use of illegal substances such as methamphetamine (METH) [3-5].

METHs are a derivative of amphetamines. Amphetamines can be used medically to treat attention deficit hyperactivity disorder, narcolepsy, and, in some cases, obesity [4,6]. Legally produced amphetamines, such as methylphenidate and phenmetrazine, may sometimes be misused recreationally [4,7]. Illegally produced amphetamines include substances such as dextroamphetamine, methcathinone, and METH [4,7]. METH (3,4‐methylenedioxy‐METH) is an illegal synthetic n‐methyl homolog of amphetamine [5], and it is a central nervous system stimulant [6]. Amphetamines and METH have a variation of effects on oral health, including broken or missing teeth, bruxism, xerostomia or dry mouth, increased risk of dental erosion, tooth surface loss, tooth‐wear, and caries [4,5]. In addition, patients taking amphetamines have an increased risk of periodontitis, mucosal ulceration, and GE [4,5].

The presence of mature dental plaque, also known as biofilm, combined with an imbalance in the host’s immune system-exacerbated by malnutrition, poor oral hygiene, hormonal changes, and lifestyle risk factors like substance abuse, can significantly impact oral and systemic health. This imbalance can lead to dysbiosis of the oral microbiome, disrupting the delicate balance of beneficial and pathogenic microorganisms [2,8-12]. Dysbiosis is characterized by a shift in the microbiome that results in a decrease in beneficial symbiotes and an increase in pathogenic microorganisms, or pathobionts [8,10,11]. As these microorganisms become more pathogenic, the host’s immune response becomes increasingly destructive [8,12]. The lack of proper oral hygiene and poor lifestyle habits favor the formation of both supra- and subgingival plaque associated with dental caries and periodontal diseases, respectively. A well-known condition related to METH abuse is “METH mouth,” which manifests as multiple caries lesions and severe periodontal problems [4,5,7]. Users with "METH mouth" often show blackened, stained, and decayed teeth, a condition thought to arise from METH-induced vasoconstriction, which impairs blood flow to the oral tissues and contributes to xerostomia (dry mouth) [4,5]. Due to these effects, METH users often consume sugary drinks to alleviate the symptoms of dry mouth [4,5]. The reduction in saliva impairs the mouth's natural defense mechanisms, including neutralizing acids produced by bacteria that metabolize sugars [11]. The high sugar content in these beverages creates an acidic environment with elevated sucrose levels, which promotes the growth of cariogenic bacteria such as *Streptococcus mutans* [10,11]. This bacterium metabolizes fermentable dietary carbohydrates, primarily sucrose, producing lactic acid as a byproduct. This acid contributes to enamel demineralization and the development of dental caries lesions [10,11]. The dysbiotic oral environment created by METH abuse also fosters the overgrowth of periodontal pathogens, such as *Porphyromonas gingivalis*, which promotes tissue destruction and contributes to the progression of severe periodontal conditions [2,10-13].

According to the most recent classification of periodontal diseases and conditions [2,13], stage 4 periodontitis is characterized by severe periodontal tissue destruction with deep periodontal pockets that may extend to the apical portion of the roots. Significant tooth mobility and a history of multiple tooth loss often complicate this stage. Grade C indicates a rapidly progressive form of the disease, often associated with systemic risk factors, such as substance abuse or severe metabolic conditions [13]. In grade C periodontitis, the rate of attachment loss is particularly high [13].

This case report presents a case of severe GE associated with a history of METH abuse in a patient with generalized stage 4, grade C periodontitis.

## Case presentation

**Figure 1:**
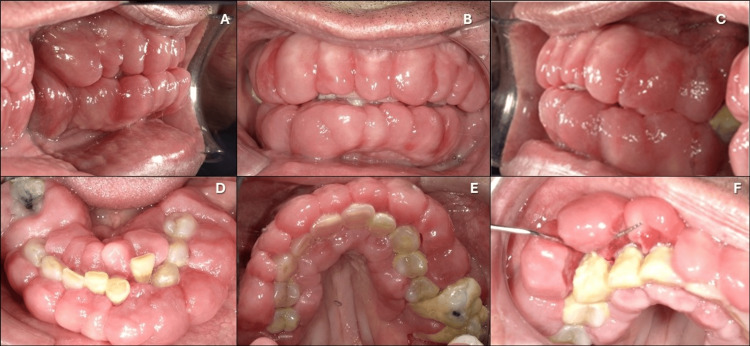
Photographs of the patient’s oral cavity displaying generalized and severe drug-influenced gingival enlargement as observed during his initial visit. (A) Right-side view. (B) Front view. (C) Left-side view. (D) Maxillary arch view. (E) Mandibular arch view. (F) Lumpy aspect of the gingiva.

A 61-year-old male presented at the LECOM's School of Dental Medicine clinic in Bradenton, Florida, United States, with the chief complaint of difficulty chewing due to enlarged, painful, and bleeding gums. His medical history included five years of use of METH, as well as a period of homelessness that resulted in poor oral hygiene. He was taking lisinopril for hypertension. The GE began two years ago and has significantly worsened over the past three months, causing considerable difficulty with eating. After receiving care at a rehabilitation facility, he was referred to our clinic upon discharge. Clinical examination revealed severe GE affecting the entire dentition, characterized by a lumpy appearance, accompanied by extensive biofilm and calculus (Figures 1A-1F).

**Figure 2:**
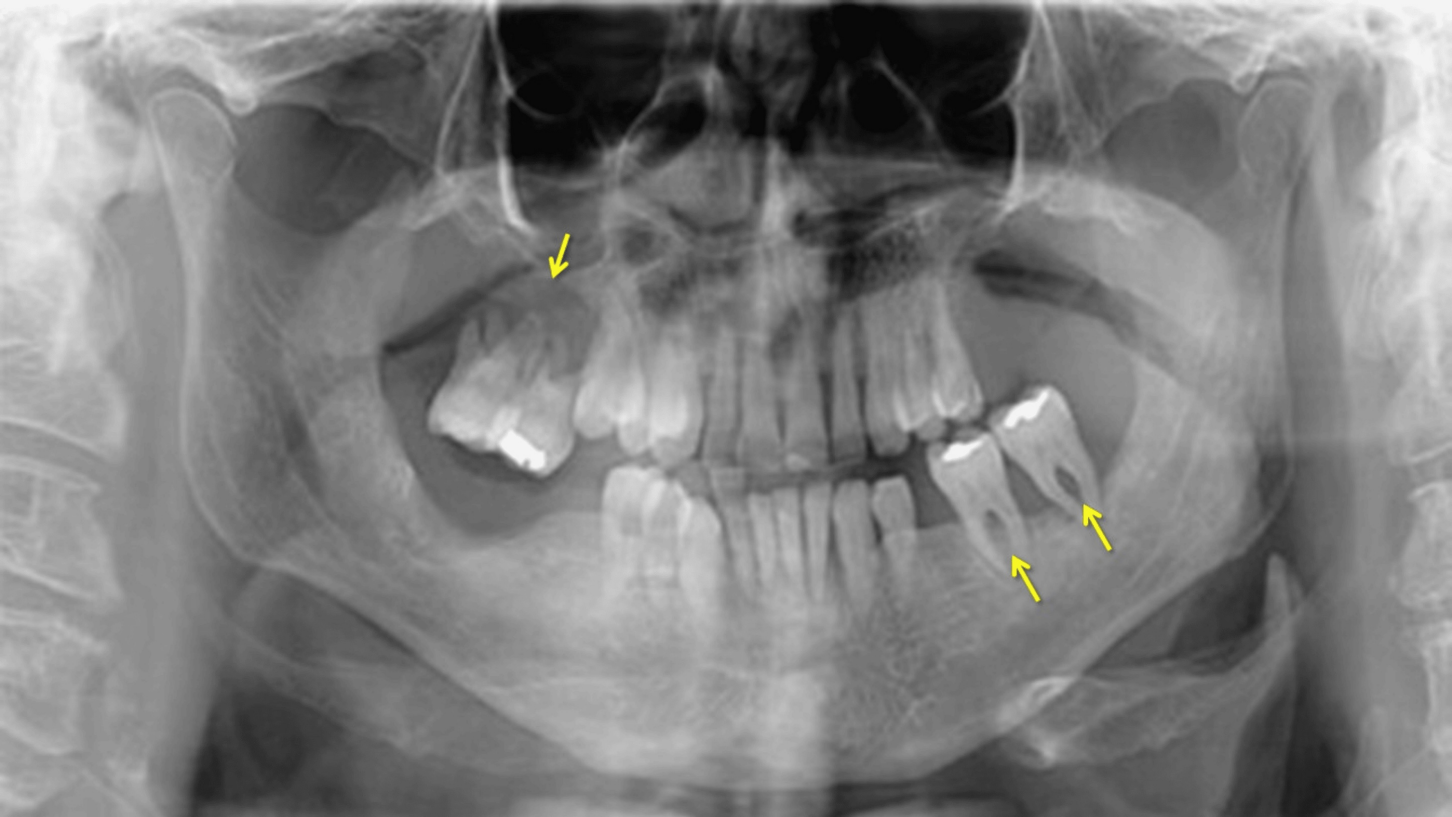
Initial panoramic radiography revealed generalized bone loss. In the remaining molars, bone loss is observed extending to the middle third of the root and beyond, with class III furcation involvement, as indicated by the arrows.

The patient exhibited spontaneous generalized gingival bleeding, deep periodontal pockets, generalized clinical attachment loss exceeding 5 mm, class 3 mobility of several teeth, and tooth loss of more than four teeth due to periodontitis. In panoramic radiography, generalized bone loss is evident. The remaining molars exhibit severe bone loss that reaches the middle third of the root and beyond, accompanied by furcation involvement (Figure 2).

Based on these findings, the patient was diagnosed with generalized stage 4, grade C periodontitis and severe drug-influenced GE.

**Figure 3:**
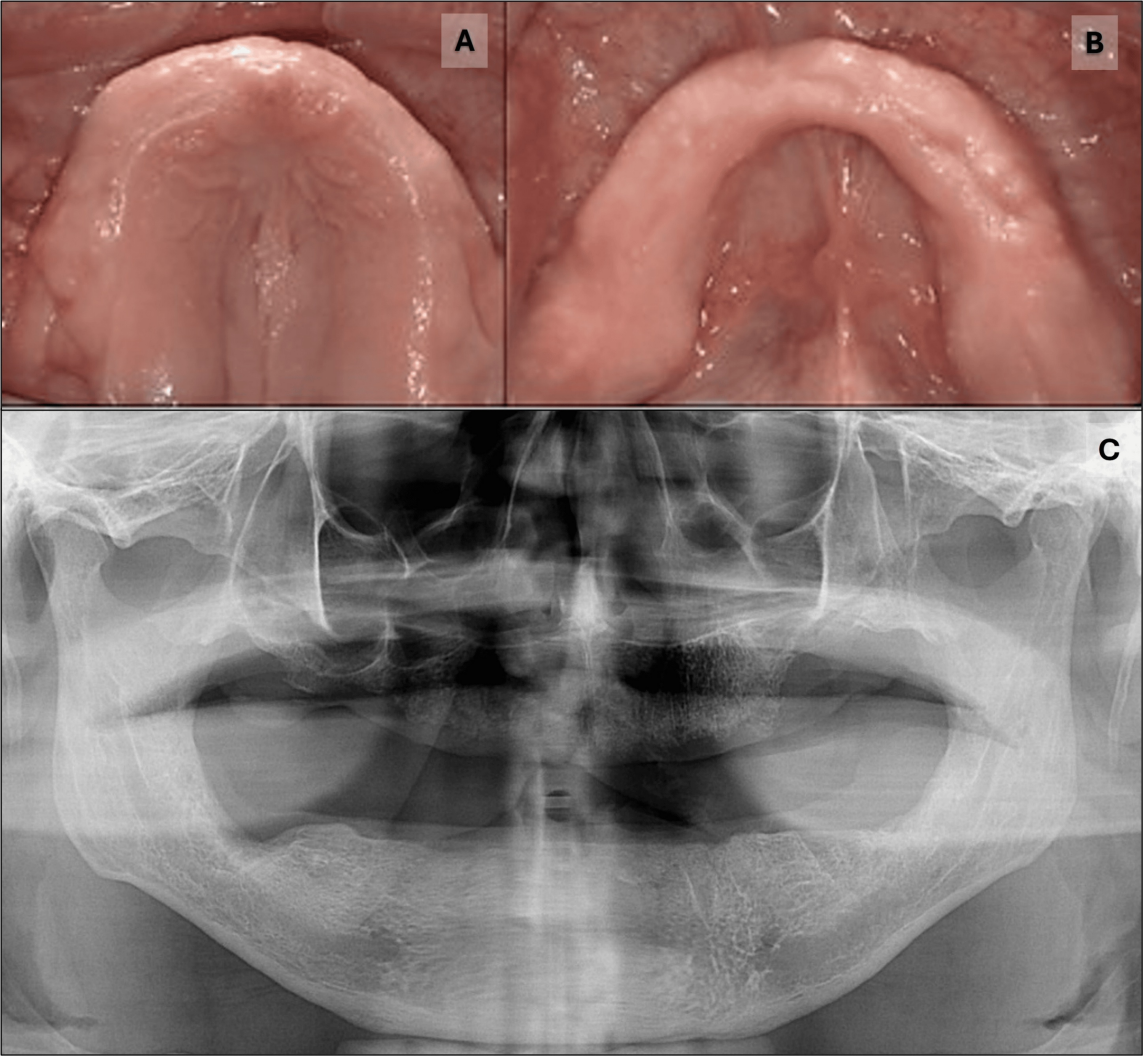
Post-operative images. (A) Maxillary arch view. (B) Mandibular arch view. (C) Panoramic radiography showing all teeth were removed.

Before surgery, the patient received antibiotic therapy consisting of amoxicillin 500 mg and metronidazole 500 mg every eight hours for 7 days, starting three days before the extractions of the remaining teeth. Standard pre-procedural protocols were followed, including assessing vital signs and using 0.12% chlorhexidine gluconate mouth rinse. Under local anesthesia, the buccal gingival overgrowth tissue was excised using a #15 scalpel blade while carefully preserving keratinized tissue to expose the dentition. Subsequent tooth extractions were performed due to the advanced periodontal disease and associated loss of supporting structures. Lingual gingival overgrowth tissue was also excised, and osteoplasty was performed with an electric high-speed handpiece using copious irrigation to reshape the underlying bone following the tooth extractions. Alveolar ridge preservation was performed at strategic extraction sites for future oral rehabilitation, potentially involving dental implants. Demineralized freeze-dried bone allograft (OsteOSS®, Hiossen, Inc., Suwanee, Georgia, United States) was used to fill the sockets, and dense polytetrafluoroethylene (d-PTFE, Osteogenics Biomedical, Inc., Lubbock, Texas, United States) membranes were placed over the graft material and intentionally left exposed to encourage the formation of attached keratinized tissue. PTFE 4-0 sutures were used. The surgeries were performed in four separate sessions, 15 days apart. The healing process was uneventful (Figures 3A, 3B). A final panoramic radiograph was taken to observe the outcomes of the surgery (Figure 3C).

**Figure 4:**
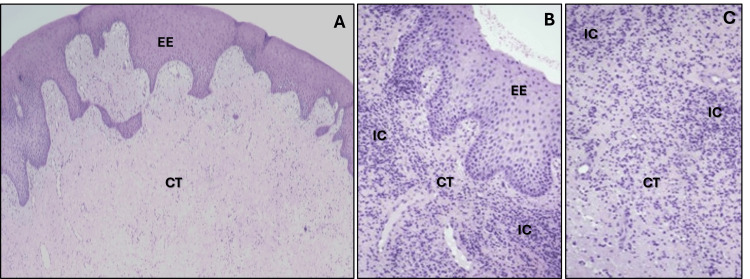
Microscopic examination of the soft tissue samples. In sample 1, the posterior upper right area (A and B) revealed keratinized stratified squamous epithelium (EE) overlying a large nodular mass of dense fibrous connective tissue. The EE exhibited a thickened layer of ortho- and parakeratin, with typical maturation patterns. In image B, the underlying fibrous connective tissue (CT) comprises dense bundles of intertwining collagen fibers interspersed with fibroblasts and occasional blood vessels, with inflammatory cells (ICs) present. Sample 2—the posterior upper right inner area (C)—exhibited similar features to those noted in sample 1.

During the first surgery, two biopsies were collected: one from the posterior upper right area of the GE (sample 1) and another from the posterior upper right inner area near the sinus floor (sample 2). These samples were sent to the University of Florida College of Dentistry, Oral and Maxillofacial Pathology Laboratory for histopathological evaluation. Microscopic examination of the soft tissue samples revealed keratinized stratified squamous epithelium overlying a large nodular mass of dense fibrous connective tissue (Figures 4A, 4B). The epithelium exhibited a thickened layer of ortho- and parakeratin with typical maturation patterns (Figures 4A, 4B). The underlying fibrous connective tissue comprised dense bundles of intertwining collagen fibers interspersed with fibroblasts and occasional blood vessels, with inflammatory cells present (Figures 4B, 4C).

The surgery's outcomes were positive, with uneventful healing. The patient initially suffered from significant pain and bleeding due to severe gingival overgrowth, which severely impacted his ability to eat and maintain proper nutrition. Following recovery from surgeries, he reported immediate relief from both pain and bleeding, allowing him to eat more comfortably, primarily with softer food textures, as he is currently edentulous. While undergoing oral rehabilitation, he looks forward to gradually diversifying his diet as his healing progresses and restorative treatments are implemented. With the burden of painful bleeding gingiva alleviated, the patient has experienced notable improvements in both his dietary intake and overall quality of life.

## Discussion

This case highlights the complex interplay between inadequate oral hygiene, biofilm accumulation, and substance abuse in the development of severe periodontitis and drug-influenced GE. The patient, a 61-year-old male, presented with significant gingival overgrowth and associated discomfort, which significantly impacted his ability to chew. His condition underscores the critical role of both local and systemic factors in the progression of periodontal disease. The patient’s period of homelessness adversely affected his oral hygiene and overall health, while reported METH use further intensified his periodontal condition.

It is well-known that biofilm accumulation is the major contributor to gingivitis [2,14] and periodontitis [1,2,11,13]. According to the polymicrobial synergy and dysbiosis model [10,11], periodontitis is driven by the mutually reinforcing interactions between a dysbiotic microbial community and an exaggerated host inflammatory response [11]. Therefore, the development, severity, and extent of periodontal diseases are influenced by a complex interplay of local and systemic factors that disrupt the balance of the oral microbiota, causing what is known as dysbiosis [8,10,12]. Poor oral hygiene, when combined with systemic risk factors, can compromise the immune system and alter the body’s inflammatory response to dental plaque [8,10,11]. Systemic factors such as substance abuse, nutritional deficiencies, chronic stress, and significant physiological changes can exacerbate periodontal inflammation [2]. Inflammation plays a key role in altering the microbial population structure associated with periodontal disease progression. The inflammatory environment exerts selective pressure on microbial community organization by supplying nutrients from tissue breakdown, which supports the growth of inflammophilic pathobionts (*e.g., Porphyromonas gingivalis, Tannerella forsythia, Treponema denticola*). These pathobionts take advantage of the altered ecological conditions to grow and intensify inflammatory tissue damage [11].

Individuals experiencing homelessness face severe challenges in maintaining good oral and systemic health due to limited access to dental care, poor nutrition, and high levels of stress [15]. The patient, having experienced a period of homelessness and substance abuse, suffered from inadequate oral hygiene, poor nutrition, and overall compromised physical and mental health [5]. These circumstances are known to weaken immune defenses and alter the oral microbiota from symbiotic bacteria to pathobionts [2,8,11]. In addition, the abuse of the use of METH aggravates the immune system weakness [4,5,7] and further exacerbates periodontal inflammation and periodontitis progression [2,11,15].

METH is a potent stimulant that exerts its effects by increasing the release of norepinephrine, dopamine, and serotonin in the brain, mimicking the activation of the sympathetic nervous system [4-6]. Specifically, METH triggers the release of norepinephrine, which binds to alpha-adrenergic receptors on salivary glands, inhibiting saliva production. The resulting decrease in saliva flow contributes to xerostomia (e.g., dry mouth), creating an environment conducive to caries lesions and oral infections [4-6]. The release of norepinephrine also induces vasoconstriction, resulting in reduced blood flow to oral tissues and impairing their ability to heal and fight off infection [5,6]. This diminished circulation worsens periodontal disease and slows tissue regeneration, making it more difficult for the periodontal tissues to recover from damage [4-6]. 

METHs also disrupt metabolic and neuroendocrine regulation, leading to improper calorie consumption and impaired nutrient processing [4,5], and those factors are known to accelerate the development and progression of GE [3,4]. Dysbiosis in the oral microbiota can promote the growth of inflammophilic pathobionts, which are highly virulent bacteria and considered major contributors to the progression of periodontal disease [2,10,11,13]. In our patient, the combination of METH abuse, poor oral hygiene, and a compromised immune system led to dysbiosis of the oral microbiota, promoting the growth of pathogenic oral biofilms enriched with inflammophilic pathobionts [2,5,8,11-13]. This dysbiotic shift contributed to GE, which progressively impaired chewing function, caused significant pain and bleeding, and resulted in excessive keratinized tissue covering the teeth.

The diagnosis of generalized stage 4, grade C periodontitis with severe GE in this patient reflects a severe form of periodontal disease [13]. Stage 4 periodontitis is characterized by extensive periodontal tissue destruction, including deep periodontal pockets, clinical attachment loss of more than 5 mm, and masticatory dysfunction. Grade C indicates a rapidly progressing disease with an accelerated rate of attachment loss of more than 2 mm over 5 years [13]. Effective management of such a severe case requires a multidisciplinary approach. Initial treatment focused on surgical intervention to remove gingival overgrown and compromised teeth, followed by alveolar ridge preservation in strategic areas and osteoplasty to prepare for future oral rehabilitation.

The patient has reported no pain while eating, which has significantly improved his quality of life. He is currently undergoing oral rehabilitation to further restore oral aesthetics and function. In addition to surgical treatment, the patient was educated on the importance of oral home care and nutrition. Regular follow-up appointments are necessary to ensure successful long-term outcomes.

To our knowledge, this is the first documented case of severe GE associated with generalized periodontitis stage 4, grade C, exacerbated by METH abuse. This case emphasizes the importance of a holistic approach to periodontal disease management, particularly when it is complicated by systemic factors such as substance abuse. Severe gingival overgrowth associated with generalized stage 4, grade C periodontitis often requires a multi-disciplinary approach, involving complex treatments such as antibiotic therapy, biopsies, extractions, gingivectomy, gingivoplasty, and osteoplasty. To achieve optimal patient outcomes, it is essential to coordinate care among a team of oral healthcare professionals, such as periodontists, dental surgeons, prosthodontists, oral pathologists, and oral microbiologists. This collaborative approach ensures a correct diagnosis and a comprehensive, tailored treatment plan that addresses the patient's complex needs. In addition to GE, METH use can contribute to other significant oral health issues, such as xerostomia and an increased risk of dental caries. Therefore, clinicians must account for the extensive effects of METH on oral tissues when developing a treatment plan. Patient education plays a pivotal role in care, emphasizing the importance of proper oral hygiene, dietary modifications, and lifestyle changes. Additionally, our patient is receiving drug abuse counseling, which is vital for enhancing mental, oral, and overall physical health. These comprehensive strategies are crucial not only for preventing further damage and managing current oral health issues but also for promoting long-term oral and overall well-being.

## Conclusions

The combination of poor oral hygiene, METH use, and associated systemic conditions contributed to oral dysbiosis, resulting in generalized stage 4, grade C periodontitis and severe GE. Comprehensive treatment included antibiotic therapy, extractions, gingivectomy, gingivoplasty, and osteoplasty to prepare for future oral rehabilitation. Patient education focused on addressing his oral health issues and providing strategies for prevention. Post-treatment, the patient reported no pain and improved eating habits, leading to a significant improvement in his overall quality of life.
